# The “Gift” of Time: Documenting Faculty Decisions to Stop the Tenure Clock During a Pandemic

**DOI:** 10.1007/s10755-022-09603-y

**Published:** 2022-06-28

**Authors:** Jessi L. Smith, L. Lynn Vidler, Michele S. Moses

**Affiliations:** 1 University of Colorado Colorado Springs, 1420 Austin Bluffs Pkwy, Colorado Springs, CO 80918 USA; 2 University of Colorado Boulder, Campus Box 049, Boulder, CO 80903 USA

**Keywords:** Work-family, Research, Pandemic, Academia, Tenure, clock-stop

## Abstract

Anticipating the deleterious effects of pandemic mitigation protocols on faculty’s research and creative work, many universities introduced mechanisms for pre-tenured faculty to receive tenure clock extensions. Unlike most stop-the-clock extensions, which occur on an individual basis, the stop-the-tenure-clock during COVID-19 was a mass-triggering event that applied to all faculty. Informed by social role theory, we examined this unique situation of stop-the-tenure clock decisions by faculty at two different universities within the same state system. Institutional level demographic and field of study data on faculty decision making at one high research activity university (n = 97) and one very high research activity university (n = 387) were examined at two time points; a first tenure-clock stop opportunity and a second tenure-clock stop opportunity. Results show that although the overall rates of clock-stops were much larger at the research-intense university, the characteristics of who was most likely to accept or opt out of the first tenure-clock stop were similar at both universities. Ethnic minoritized faculty at both universities had greater odds of accepting the clock-stop. Results also showed that at both universities, women were somewhat more likely to accept the first tenure clock extension, and exploratory follow-up shows this gendered decision manifested differently depending on field of study. Relatively few faculty accepted the second tenure clock-stop. Our findings provide a portrait of who accepts or declines tenure clock extensions with important implications for downstream effects on equity within the academy.

The COVID-19 pandemic put a magnifying glass on the gendered and racialized nature of work-life imbalance (Blundell et al., 2020; Lechuga-Pena, 2021; Powell, 2021; Reese et al., 2021) and allowed for an examination of workplace practices meant to support people during life-altering events. The pandemic “upended almost every facet of academia” (Malisch et al., 2020) from economic disruptions, technological challenges, and communication struggles to new cleaning protocols, compliance concerns, and strains on human-resource management. The negative impact was felt in all facets of university life.

Mitigation efforts, such as the closure of schools, daycares, and senior assisted living centers, created unprecedented domestic demands on people’s, especially women’s, time. One survey showed that during the height of the pandemic, parents spent upwards of 9 h a day on childcare and 3 h a day on housework (Andrew et al., 2020). The gendered division of caregiving labor is not new and is central to the tenets of social role theory (e.g., Eagly & Wood, 1991; Eagly, Wood, & Diekman, 2000), which posits that the historical frequency of men and women in different role positions (e.g., as provider versus caregiver) has created norms for gendered and racialized behaviors (e.g., men as leaders and woman as teachers) that then reproduce the status quo of gender and racial distributions. The ways in which such social roles clash with the structures of the academy and contribute to work-life “conflict” for women and people of color is well-established within the literature (e.g., Deutsch & Yao, 2014; Lechuga-Pena, 2021; Mason et al., 2013; Moors et al., 2014; Powell, 2021), but the disproportionate impact of caregiving on women’s time is greater than before the pandemic (Andrew et al., 2020; Langin, 2021). Added to this toll is the expected and actual emotional labor that women, especially women of color, perform to care for others (e.g., Erickson, 2005), including tending to the relational needs of family and co-workers (e.g., Brummelhuis & Greenhaus, 2019; Williams & Dempsey, 2014).

The pandemic necessitated a reprioritization in faculty workload. Most faculty rapidly moved their teaching and mentoring activities online and adapted to remote learning. Access to libraries was limited, and in many cases interlibrary loan operations were unavailable. With rare exception, faculty were not permitted to access their research or studio equipment, data collection and creative performances often were halted, and travel to field sites and conferences was restricted. The pandemic sent most faculty to makeshift home offices to find ways to engage virtually with their students and colleagues, which was both frustrating and mentally taxing (Ramlo, 2021). Advancing a research agenda became secondary to the primary goal of serving students. As Inouye et al. (2020) put it, “pandemic-induced restrictions on research are now ubiquitous” (p. 724). By one estimate, faculty reported an 11% decline in the time they devoted to their work because of COVID-19 (Myers et al., 2020). In another survey commissioned by the NIH of over 45,000 researchers, findings demonstrated that laboratory-based researchers and early-career researchers reported the greatest concerns regarding disruptions and negative impacts of COVID-19 on their careers (Bernard & Lauer, 2021).

As might be predicted from social role theory (Eagly et al., 2000) women’s research productivity took a particularly hard hit (e.g., Barber et al., 2021; National Academies of Sciences, Engineering, and Medicine, 2021) likely because of their primary caregiving roles (Williams & Dempsey, 2014). During this time, women across all fields submitted significantly fewer manuscripts for peer review compared to men and fewer compared to women’s submission rates pre-pandemic (Squazzoni et al., 2020; Ribarovska et al., 2021). Women, especially those with young dependents, reported a 17% larger decline than men in time available to conduct research, when everything else was held constant (e.g., career stage, access to campus during the lockdown, field of study) (Myers et al., 2020). Women-identified faculty also started fewer research projects and completed on-going projects at slower rates during COVID-19, compared to the same months in 2019 (Viglione, 2020). Research on finance scholars’ productivity showed that, in addition to spending time with young children, women were more likely to spend more time teaching, which also negatively affected their research output (Barber et al., 2021).

Women scholars in the U.S. were also less likely to participate in the rapid research related to COVID-19 itself (e.g., Ribarovska et al., 2021). For example, a review of 1,893 medical journal showed that the proportion of women leading the science on COVID-19 (in a range of fields) and taking the first-author role was significantly less compared to the proportion of women publishing in the same journal the year before (Andersen et al., 2020). To conduct research on the novel coronavirus required the ability to reprioritize a program of research, receive funding, and quickly implement protocols, analyze data, and write up findings for fast-tracked peer review. Andersen et al.’s results not only suggest that women were less likely to participate as leading scholars in this process, but also they also undergird the lack of diverse perspectives in understanding the biomedical aspects of COVID-19 (Silver, 2019).

Research and creative productivity is often at the heart of promotion and tenure reviews. As the American Association of University Professors summarizes, the purpose of tenure is to “safeguard academic freedom, which is necessary for all who teach and conduct research in higher education. When faculty members can lose their positions because of their speech, publications, or research findings, they cannot properly fulfill their core responsibilities to advance and transmit knowledge.” (AAUP, 2020). Women and faculty of color are underrepresented as tenured professors (NSF, 2021). Given the high-stakes nature of tenure, many institutions of higher education responded to the pandemic by enacting automatic stop-the-clock tenure policies to extend the probationary period for one year for all pre-tenure faculty (Myers et al., 2020; Pettit, 2020). Such policies permit faculty to stop the tenure clock for major life events (e.g., birth or adoption of a child, caring for an ill partner) or events outside the faculty member’s control (e.g., unforeseen obstacles to setting up a research lab). The best practice is to make stop-the-clock policies automatic, so that faculty do not need to take any special action or experience any additional burden during their vulnerable time; however, anyone can “opt out” and decline to stop their tenure clock (Williams & Lee, 2016). Such opt-out policies help prevent “flexibility stigma” (Cech & Blair-Loy, 2014) that comes when someone is devalued or marginalized for needing modified duties.

## Study Overview

Pandemic tenure extensions are a concerted effort to ease the burden on faculty suffering significant obstacles to research productivity (Malisch et al., 2020). On the one hand, social role theory (and the available evidence related to research productivity reviewed above) would predict that women faculty and faculty of color would have the greatest need to stop-the-tenure-clock because of their conflicting caretaking obligations during the pandemic. On the other hand, some research suggests that this “gender and race-neutral” policy, can unintentionally benefit those who are already privileged (e.g., white men) (Antecol et al., 2018). That said, unlike most stop-the-clock extensions which occur on an individual basis depending on a life event, the stop-the-tenure-clock during COVID-19 was a mass-triggering event that applied to all faculty. Such an event therefore presents an important opportunity for academia to look inward and examine the use of newly enacted stop-the-clock policies to prepare for possible long-term implications.

Our aim was to test whether and how stop-the-clock decisions differ as a function of the university’s research intensity, faculty career stage, and faculty’s gender and racial identity. We hypothesized that in general, faculty at the R1 university and those faculty earlier in their career stage would be more likely to keep the clock-stop. Based in social role theory, we further hypothesized that women and faculty of color would be especially likely to keep the clock-stop and this would be true at both universities. We also wanted to explore possible trends in any gendered decisions as a function of academic field. Finally, we describe the pattern of decisions made by faculty regarding the second clock stop option. We used a combination of inferential and descriptive analyses to answer our research questions with available institutional level data by faculty at two different universities within the same state system.

## Methodology

### Two University Contexts

Wanting to set an example for data-informed planning and introspection, we collected institutional level data from our two universities within the same state system (we did not collect data from the other campuses, one of which is a medical school). The advantage of a descriptive study of the two populations of convenience was that we could examine stop-the-clock use within a “high research activity” regional comprehensive university (an R2) and a “very high research activity” flagship research university (an R1) serving the same state. Once all the data were coded (outlined below), the data were scrubbed for names and any personal identifying information. The Institutional Review Board approved this study.

The *R2 University* serves 7 colleges and approximately 12,000 undergraduate students, 2000 graduate students, and 288 tenured/tenure-track faculty with 53 baccalaureate, 24 master’s, and 8 doctoral degree programs across a broad range of disciplines. The STEM fields represented at the R2 include Biology, Chemistry & Biochemistry, Computer Science, Electrical and Computer Engineering, Geography and Environmental Studies, Mathematics, Mechanical & Aerospace Engineering, and Physics & Energy Science. The social and behavioral science fields (SBS) included Anthropology, Economics, Political Science, Psychology, and Sociology. The humanities and creative art fields include Visual and Performing Arts, Communications, English, History, Languages & Cultures, Philosophy, and Women’s and Ethnic Studies. The professional fields include Education, Counseling, Business, Nursing and Health Sciences, and Public Affairs.

The *R1 University* is one of 36 U.S. public research institutions in the Association of American Universities and has approximately 1,300 tenured and tenure-track faculty across 7 schools and colleges, 12 research institutes, and university libraries, serving 29,000 undergraduates and 6,000 graduate students. The fields represented at the R1 were similarly categorized as the R2 University into STEM (e.g., Astrophysical & Planetary Science, Applied Mathematics, Computer Science), SBS (e.g., Economics, Environmental Studies, Sociology), Professional (e.g., Business, Education, Law) and Humanities/Arts (e.g., History, Philosophy, Theatre & Dance).

### First Stop-the-clock Process for COVID-19

Discussions regarding the first tenure-clock stop began very early on in the pandemic response at both universities, approximately mid-March 2020. The campus announcement at both universities emphasized that the clock-stop was *automatic* for all pre-tenured faculty with details on how a faculty could choose to opt out of the tenure clock stoppage (and, for example, go up “early” on their previous tenure clock schedule).

### Second Stop-the-clock Process for COVID-19

The process for a second stop-the-clock in 2021 diverged at the two universities. In spring 2021, as the vaccine roll-out was at its height, The R2 university approved a second tenure-clock stop, which permitted faculty to delay tenure review for two full years. This stop was automatic for all tenure-track faculty at the R2 and included the option both to opt out, and/or to reverse the first clock-stop decision. New pre-tenured faculty made the decision for the first time. A few tenure-track faculty (n = 8) who were ineligible for the first clock-stop (because they were already on an approved clock-stop for non-COVID reasons) also became newly eligible.

At the R1 university, a second clock stoppage required an “opt in” request to the chair, director, or dean. The supervisor then informed the appropriate faculty human resources liaison who administers tenure reviews for the unit. The faculty liaison then submitted the Optional Second Tenure Clock Stoppage Request form to the Office of Faculty Affairs, so the faculty member’s tenure clock dates could be adjusted officially in their personnel records. No deadline was given, and faculty can (still) request the second tenure clock stop up until the time of their review.

### Participants and Procedure

#### First Clock Stop R2 Population

Data at the R2 university were obtained on the clock-stop decisions made by pre-tenured faculty in 2020 (n = 98 faculty) One faculty member voluntarily left the university, resulting in a total of 97. Faculty who stopped the clock for non-COVID reasons (n = 8) were included in analyses and coded as having stopped the clock.

The IR Office compiled information from human resource data sources about the person’s self-identified demographics (gender, race/ethnicity) and years of service at the university. The years-of-service data included the original start date of the individual. For more than 20% of the sample (n = 21), this included a previous affiliation with the university (e.g., as a former non-tenure-track instructor or as a former student employee). Data could not be obtained as to the actual “years-to-tenure” as a pre-tenured faculty member for this group; as such, for anyone with more than 7 years of service, this variable was treated as missing. Although not ideal, we subtracted 7 from the years of service given that faculty go through promotion and tenure review during the seventh year. This new variable was treated as the R2 university’s years-to-tenure variable.

Faculty self-identify their gender and race/ethnicity in their employee portal, which IR accessed for reporting. Non-binary gender designations are not permitted in the current HR system. Of the final 97 faculty, 51.5% were identified as women, 48.5% as men. Furthermore, 73.2% were identified as White or Asian, 10.3% as racially or ethnically minoritized (i.e., American Indian, Black, Hispanic, or multiple racial identities), and 4.1% as international/non-residents. 12.4% were unknown with missing data.

#### First Clock Stop R1 Population

Data at the R1 university were obtained on the clock-stop decisions made by pre-tenured faculty (n = 387 faculty) on whether they chose to “opt out” (by going up early, on their original schedule) for the initial tenure-clock stop. Human resource data were again used to categorize the person’s self-identified demographics (gender, race/ethnicity) and years of service at the university. The years-of-service data included the original start date of the individual. Like the R2 university, faculty are asked to self-identify their gender and race/ethnicity in their employee portal with no option to select a non-binary gender designation. Of the 387 faculty, 46.3% identified as women, 53.7% as men. Furthermore, 64.1% identified as White or Asian descent, 13.4% as racially or ethnically minoritized (i.e., American Indian, Black, Hispanic, or multiple racial identities) and 22.5% did not specify.

#### Second Clock Stop Population

Data from the R2 university were collected in a similar manner as the first wave of data, with two primary differences: (1) data included the second clock-stop decision as well as the decision on changing the first clock-stop decision and (2) years of service was not collected. Data were collected from all pre-tenure faculty (n = 95). Faculty who stopped the clock for non-COVID reasons (n = 1) were included in analyses and coded as having stopped the clock. Faculty hired in 2020 (n = 13) were for the first time deciding to keep the automatic stop or opt out. Faculty who went up for tenure and promotion in 2021 (n = 9) were not eligible for the second clock stop but were coded to determine whether they changed their first clock stop decision from 2020. All told, n = 76 faculty made second clock stop choices, n = 83 also decided whether to change their first clock-stop choice, and n = 13 were new hires making their first clock-stop decision.

Of the 95 R2 faculty, 47.4% identified as women, 52.6% as men and 75.8% identified as White or Asian, 13.7% as racially or ethnically minoritized (i.e., American Indian, Black, Hispanic, or multiple racial identities), and 4.2% as international/non-residents and 6.3% did not specify. 

Because the process for requesting the second tenure clock stop is not time bound at the R1 university, 6 months following the announcement of the option we collected data only on those who requested to opt-in.

## Results

### Analysis Overview

We used logistic regression to test whether and how stop-the-clock decisions differed as a function of the university’s research intensity, faculty career stage, and faculty’s gender and racial identity. Specifically, we conducted a stepwise logistic regression analysis with stop-the-clock decisions (0 = no, declined the clock stop; 1 = yes, accepted the clock stop) as the dependent variable and the categorical independent variables of university (R2 = 0; R1 = 1), gender, (men = 0; women = 1) ethnicity (Majority/Unknown = 0; Minoritized = 1) and the continuous independent variable of years-to-tenure-review. We did not include field of study because of the disparities in the representation of gender and minorities within fields and the uncertainty with which field to set as the comparison. We therefore paired the logistic regression with Chi-Square as a nonparametric test to further unpack overall trends in decisions about stopping the tenure clock for COVID and how these trends were associated with faculty gender and field of study at each university. In this way, we used both inferential and descriptive statistics to test our question about the nature of gendered decision-making patterns in stop-clock decisions.

#### First Clock Stop Decisions

Table 1 shows logistic regression results. As shown, faculty at the R1 university were more likely to keep the tenure clock stop compared to faculty at the R2 university, with an odds-ratio = 4.294. The odds-ratio for faculty gender (1.314) indicated that women-identified faculty at both universities were marginally more likely to keep the tenure clock stop compared to men-identified faculty, though the odds ratio was greater than one, this gender difference was not significant. Results also showed that ethnically minoritized faculty at both universities were slightly more likely to keep the tenure clock stop compared to majority faculty (odds ratio = 1.728); the odds ratio was greater than one, but was not significant. Finally, results illustrated that faculty closer in time to their original tenure review were significantly less likely to want to stop the tenure clock (odds ratio = 1.873).

**Table 1:** Logistic regression model predicting faculty decision to accept the first tenure clock stop for COVID-19

Factor	Β	SE	Wald (df)	p	Exp Β	95% CI for EXP Β Lower Upper			
University	1.46	0.29	25.34 (1)	0.00	4.29		2.43	7.57	
Gender Identity	0.27	0.25	1.24 (1)	0.26	1.31		0.81	2.12	
Ethnic Identity	0.55	0.40	1.87 (1)	0.17	1.73		0.79	3.79	
Years until tenure review	0.63	0.08	58.89 (1)	0.00	1.87		1.60	2.20	

#### Examining Trends in Gendered Decisions at Each University

Given the significant difference by type of university, we drilled down to examine the descriptive statistics within the R2 and R1 university separately. Results showed that within the R2 university, 48.5% (n = 47) of pre-tenured faculty accepted the COVID tenure-clock extension and 51.5% (n = 50) declined. Of those who accepted the extension, 57.4% identified as women and 42.6% identified as men. Of those who declined, 46% identified as women and 54% identified as men (see Table 2). Calculating the odds ratio for women who accepted the clock-stop (57.4/46 = 1.248) and the odds ratio for men (42.6/54 = 0.788) and comparing them (1.248/0.788) indicate that, relative their population on campus, women-identified faculty at the R2 university were one-and-a-half times more likely (odds ratio = 1.59) to accept the tenure-clock extension than men-identified faculty.

Analyses within the R1 university showed that 79.3% (n = 307) of pre-tenured faculty accepted the tenure clock stop for COVID and 20.7% (n = 80) declined the extension and chose to go up “early” (on their original timeline). Of those who accepted the tenure clock stop, 47.6% identified as women and 52.4% identified as men. Of those who opted out of the clock stop, 41.3% identified as women and 58.8% identified as men (see Table 2). Using the same odds ratio formula as above, these results indicate that, relative to the population on campus, women-identified faculty were almost one-and-a-half times more likely (odds ratio = 1.30) to keep the tenure-clock stoppage than men-identified faculty.

#### Examining Trends in Gendered Decisions at Each University with Type of Field

We used Chi-Square as a nonparametric test to compare frequency counts to test if decisions about stopping the tenure clock for COVID were associated with faculty gender and field of study at each university. Results showed a meaningful difference of these factors at both the R2 university *X*^2^ (3, n = 97) = 10.10, *p* < .02 and at the R1 university *X*^2^ (3, n = 387) = 12.44, *p* < .01.

Table 2 illustrates the R2 university data and shows that, among women-identified faculty in SBS fields, 100% accepted the tenure-clock extension whereas those in the humanities had the greatest percentage who opted out (37.5% accepted the clock stop, 62.5% opted out). In contrast, among men identified faculty, 100% declined the extension with SBS fields and those within STEM fields had the greatest frequency of accepting the extension (68.8% accepted, 31.3% declined). Within the professional fields, both men and women-identified faculty tended to opt out.

Table 3 illustrates the R1 university data and shows that in all fields, men- and women-identified faculty were more likely than not to accept the tenure-clock stop. Similar to the R2 results, among women-identified faculty, those within SBS fields had the greatest percentage of accepting the clock stop (94.1% accepted, 5.9% opted out) and those within humanities/art fields had the greater percent of opting out (78.7% accepted, 21.3% opted out). The reverse pattern emerged for men-identified faculty, whereby those within humanities/art fields were most likely to accept the extension (86.7% accepted clock stop, 13.3% opted out) whereas those within SBS fields had the greatest percent of opting out (68.2% accepted, 31.8% opted out). Within the professional fields and STEM fields, both men and women-identified faculty, more often than not, accepted the clock-stop.

**Table 2:** Descriptive statistics for faculty at a high research activity (R2) university who did and did not accept the first tenure-clock extension for COVID-19

First Stop-the-Clock Decision		STEM N, %	SBS N, %	Professional N, %	Hum/Arts N, %	Total N, %
Declined: did not stop the clock	Women	5 45.5%	0 0%	13 59.1%	5 62.5%	23 46%
	Men	5 31.3%	6 100%	12 63.2%	4 66.7%	27 57.4%
	Ethnically Minoritized					2 20%
	International					2 50%
Accepted: stopped the clock	Women	6 54.5%	9 100%	9 40.9%	3 37.5%	27 54%
	Men	11, 68.8%	0, 0%	7, 36.8%	2, 33.3%	20, 42.6%
	Ethnically Minoritized					8 80%
	International					2 50%

*Note*. Frequency counts and percent within gender identity by field are displayed. STEM = science, technology, engineering, math; SBS = social behavioral sciences; Hum/Arts = humanities and arts

**Table 3:** Descriptive statistics for faculty at a very high research activity (R1) university who did and did not accept the first tenure clock extension for COVID-19

Stop-the-Clock Decision		STEM N, %	SBS N, %	Professional N, %	Hum/Arts N, %	Total N, %
Declined: did not stop the clock	Women	13 19.7%	1 5.9%	9 18.4%	10 21.3%	33 18.4%
	Men	27 25%	7 31.8%	9 18.75%	4 13.3%	47 22.6%
	Ethnically Minoritized					9 17.3%
Accepted: stopped the clock	Women	53 80.3%	16 94.1%	40 81.6%	37 78.7%	146 81.6%
	Men	81 75%	15 68.2%	39 81.25%	26 86.7%	161, 77.4%
	Ethnically Minoritized					43 82.7%

*Note*. Frequency counts and percent within gender identity by field are displayed. STEM = science, technology, engineering, math; SBS = social behavioral sciences; Hum/Arts = humanities and arts

#### Second Clock Stop Decisions

Logistic regression results did not yield any significant effects of any of the variables predicting the decision to accept the second clock-stop. Drilling down into each university showed that only a small proportion of faculty stopped their clock twice. At the R2 university, 38.2% (n = 29) of pre-tenured faculty accepted the second COVID extension and 61.8% (n = 47) declined and of those who accepted the extension, 48% identified as women and 52% identified as men. Of those who opted out of the second clock stop, 49% identified as women and 51% identified as men.

Six months following the announcement of the option at the R1 university (which did not set a due date), a total of n = 24 faculty had requested the second stop, and all were approved. Of those opting into the second tenure clock stop, 37.5% identified as women and 62.5% as men.

#### First Clock Stop Decisions Revisited

The R2 university offered faculty the chance to change their minds about their first clock-stop and analyses showed that 96.4% (n = 80) of faculty did not change their first clock decision; among those who did change their first decision (n = 3, 3.6%, 2 men-identified and 1 woman-identified faculty) all chose to reset their clock to the original timeline. Sample sizes were too small to detect any statistical differences.

#### New Hire First Clock Stop Decisions

The R2 university also offered new hires the chance to stop their tenure clock for the first time. Analyses showed that 31% (n = 4) of pre-tenured faculty making their first stop the clock decision in 2021 accepted the clock stop, whereas 69% (n = 9) declined. Of those who accepted the clock stop, 100% were men whereas among those who opted out 33.3% were men-identified and 66.7% were woman-identified. The sample size was again too small to detect statistical differences and should be considered only as exploratory.

## General Discussion

Though the two universities represent very different research contexts (R2 and R1), the pattern of stop-the-tenure-clock decisions both diverged in meaningful ways and shared overlap. Results indicated that the research-intensity of the university was one important difference in the overall decision to stop the tenure clock. At the high-research-activity (R2) university, about half of pre-tenured faculty accepted the initial tenure-clock extension and about half declined. In contrast, at the very high research activity (R1) university almost 80% of pre-tenured faculty accepted the initial tenure clock stop.

Though the overall rates of acceptance were much larger at the research-intense university, the characteristics of who was most likely to accept or opt out of the clock-stop was similar at both universities. The data show that ethnically minoritized faculty at both universities had greater odds of accepting the tenure clock extension. The decision to accept the initial extension differed somewhat by faculty gender, though not as clear-cut as social role theory might predict. Descriptive analyses suggests that this gendered decision differed depending on field of study. Compared to men-identified faculty, women-identified faculty in SBS at both universities had greater percentages of accepting the tenure-clock stop. Women in the humanities had greater rates of opting-out and keeping their original timeline. No consistent pattern emerged for men-identified faculty’s decisions across fields or between the two universities. For example, at the high-research university, men in STEM had the highest percentage of stopping the tenure clock, whereas at the very high research university, men in the humanities had the highest percentage of accepting the tenure clock stop. At both universities, faculty closer in time to their original promotion and tenure review timeline were more likely to stay with that original timeline. Finally, when given the option for a second tenure-clock stop, very few faculty at either university chose to take it.

### Limitations and Future Directions

Of course, our results were limited by the available institutional data. We did not collect data on *why* faculty made their decisions; we focused on describing the characteristics of those who did and did not decline the stop-the-clock extensions offered in response to the pandemic. Our results point to needed future research on gendered and racialized experiences within different fields. For example, why might gender and racialized experiences in humanities and social behavioral science fields result in divergent decisions? Social role theory would predict that fields in which women and people of color are most underrepresented (and cis-gender white men well represented) should lead to norms and stereotypes that call for agentic, white, masculine behavior (e.g., Diekman & Eagly, 2000; Miller et al., 2015) that might deter women and faculty of color from stopping the tenure clock (compared to fields in which they are better represented where they might be more supported in making a tenure clock-stop decision). Testing this hypothesis in future research using representation data would be a welcome addition to the literature.

Our data also suggest the second stop-the-clock was not a popular option. Why would faculty be so unified in their decision to reject a second clock stop, despite two very different approaches to offering it? At the R2 university the second stop was automatic and at the R1 university it was an opt-in process. Our data illustrate one way to use institutional level data to examine an emerging problem and solution, but such data can only take us so far. Future research would do well to couple such a descriptive analysis with more robust qualitative and quantitative measures. We also do not know the full implications of faculty decisions; only longitudinal analyses will shed light on the ramifications of stopping the tenure clock (once or even twice) in response to COVID-19.

Our study’s findings reflect the increased pressure and expectations that many faculty face at high research activity institutions. We did not include non-pandemic era use of clock-stop for comparison, and future research is needed to tease out if our pattern of results is specific to the pandemic-era or more generally a reflection of field and university structures. We speculate the latter, given that tenure policies within our state system tend to favor sameness of treatment over equity, which may result in more favorable outcomes for faculty with majority identities. This brings up an important question: whether and how tenure criteria and university policies should recognize or account for diverse life circumstances of faculty? (e.g., Matthew, 2016). The time is right for institutions to consider how they can be more supportive of their faculty during trying times (in general) and how tenure policies might benefit from revision.

### Implications

There are lessons to be learned from what we do know about pre-COVID-19 pandemic “gender-neutral” tenure-clock stop policies. The state system’s tenure-clock stop policy, like many universities, grants a one-year extension of the probationary period whenever family medical leave or parental leave is taken. It also permits applications for clock-stops for other reasons. However, studies show that such “gender-neutral” clock-stop policies not only do not benefit women-identified faculty but often provide a significant advantage to men-identified faculty (Antecol et al., 2018; Smithson & Stokoe, 2005). Though men-identified faculty can and do experience work-family needs (Reddick et al., 2012), research on social role theory documents time and again that women-identified faculty report higher levels of work family demands and conflicts (Williams & Lee 2016; Powell 2021) and this is especially true for women of color (Kamenou, 2008; Williams & Dempsey, 2014). Indeed, work-life considerations are an important reason why women-identified Ph.D.s do not pursue tenure-track positions at the same rates as their men-identified counterparts (Mason et al., 2013; Settles et al., 2006). Therefore, even though many men-identified faculty members in this study accepted the tenure-clock stop, their experience and potential for progress should not be equated with the women-identified faculty members who did the same. As Antecol et al. (2018) stated, “gender-neutral clock stopping policies will not level the playing field in terms of tenure outcomes if men are able to use the extra time more productively or strategically than women” (p. 2422). We contend that when faculty issues being addressed by campus leadership are not gender-neutral, then the policies ought not to be gender-neutral either.

Stopping the tenure clock is a complicated decision with long-term ramifications. Stopping the clock delays accompanying pay raises and promotions which, over time, may compound gender and racial pay disparities (Barnum et al., 1995; Freund et al., 2016) and limit the leadership and influence opportunities that come with promotion (Britton, 2010). Stopping the tenure clock could hurt the very faculty members the policy is meant to help (Khamis-Dakwar & Hiller, 2020). So, what options does this leave for university leadership who want to be responsive?

The COVID-19 pandemic was an unforeseen crisis that demanded a university response to reduce the stress on pre-tenured faculty. Our data answer the question about who does and does not opt to stop the tenure clock at two universities and paints a portrait of social role informed gendered, racialized, and field-based “microcultures” (Thoman et al., 2017) that factor into this life-altering decision. The next step is to proactively mitigate negative, long-term, unintended consequences. Addressing the crisis takes a multipronged approach by university and shared-governance leaders to revise evaluation structures in light of the pandemic’s expected long-term devastation on universities’ budgets, enrollment, faculty productivity, and the larger community’s well-being (Galea et al., 2020; Cardel et al., 2020; Whitford 2020; Reese et al., 2021).

Where can university leadership start? Research shows that stopping the clock for family reasons may signal to reviewers that the faculty member has a lower commitment to academic work (Flaherty et al., 2013). Knowing this, one strategy is to provide reviewers with an explicit statement about the university’s concern and commitment not to penalize faculty for COVID-19 impacts (whether or not the person stopped the tenure clock). An explicit statement in the evaluation instructions could direct external and internal reviewers to consider quality, not quantity, ignore gaps in the record, and to disregard years in rank, all in favor of the overall contribution and impact of the work. This type of letter will be necessary for years to come, even when the pandemic is (hopefully) far behind us. For example:

Dear Evaluator: The COVID-19 pandemic has taken a serious toll on productivity and faculty career progress throughout higher education. {This University} seeks to be attentive to the pandemic’s extenuating circumstances as we evaluate this candidate’s case.

At {This University}, every pre-tenured faculty member was granted a one-year tenure clock stoppage, as well as the option of a second clock stoppage if needed. These clock stoppages should not be treated as “extra time” in that they should not raise tenure or promotion expectations. Rather, the clock stoppages should be understood as a way to account for the disruptions, delays, and work stoppages resulting from pandemic-related obstacles to scholarly and creative productivity.

As you consider this candidate’s professional achievements, we invite you to also consider and acknowledge the very real toll of the COVID-19 crisis and our campus’ ongoing response to the pandemic. It is reasonable to expect that the pandemic mitigation efforts such as moving to remote learning, limited access to campus research spaces and resources, and restricted travel, have and will lead to variation in the period for review. Therefore, we would appreciate that in evaluating this candidate, you do not consider the number of years since PhD or years in position. Instead, your evaluation, consistent with departmental unit criteria, should consider the quality of the work and the impact on the field in cases where the quantity, rate, or timeliness of the accomplishments were impacted by COVID-19 pandemic effects. We hope you will use an empathic assessment of research productivity, teaching effectiveness, and service commitments that acknowledges the vastly different circumstances faculty are operating under and adapting to because of the pandemic.

It might also be beneficial for faculty to provide “COVID-19 Impact Statements” that they can submit with their annual review and/or promotion materials. Such impact statements can take the form of answering pre-determined questions (see, for example, the supplemental questions in Malisch et al., 2020) or can be open-ended narrative responses that allow for personalization and nuance. Annual reviews, for years to come, should also consider the long term COVID-19 impacts – whether the faculty member stopped the tenure clock or not. To this end, typical annual review processes ought to be reimagined, for example, by enlisting the guidance of a Pandemic Response Faculty Fellow (Malisch et al., 2020). Another strategy to consider is allowing faculty to retroactively adjust their workload distribution to reflect their reality. For example, if a faculty member is typically evaluated using a 40% teaching 40% research and 20% service formula, allow that faculty member to set their own differential workload percentages for evaluation based on their experience of COVID-19. Of course, this may not be feasible at all institutions for contractual reasons. However, faculty impact statements could, at a minimum, outline a faculty member’s actual effort distribution during the performance period.

Yet another strategy is to allow faculty to revisit their decision to accept or decline the tenure-clock extension. Given the long lead time required for research productivity, a quick decision in the midst of the pandemic might not be fully informed. Allowing faculty, including faculty hired during the pandemic, to re-consider their choice would ease the burden of being locked into a decision they might regret. University leadership could also consider ways to prioritize teaching and research support to women-identified and minoritized faculty that could help offset some of the time lost to research productivity. This could include teaching assistant support, scheduling preferences, research seed grants, course off-loads, or differentiated workloads. We also suggest that department chairs and other academic leaders would benefit from crisis-leadership trainings to help them navigate the “intensified challenges” the pandemic laid bare (Gigliotti, 2021).

Finally, this study presents research universities with a values question—to what extent is it a priority for research universities to ensure a diverse professoriate, even when the pandemic is long behind us? Given the research on the educational and epistemic benefits of a diverse academy (e.g., Gurin et al., 2002; Moses, 2006), what is the moral imperative to support our minoritized faculty through whatever challenging life circumstances they may be facing? To be clear, this is not about lowering standards. But how do universities consider a holistic approach to evaluation given additional and disparate stressors and responsibilities? As we noted, gender-neutral work policies, including gender-neutral tenure-clock stops, often unintentionally widen disparities. Given this, where funds are available, we suggest that supplemental resources such as additional subsidized childcare capacity, affinity group mentorship, grants for impacted faculty, free mental healthcare and other similar opportunities be made available. These are less likely to be used by those who do not need them, and are, therefore, less likely to create additional disparities. Although there is no one magic solution that will fit all situations, it is essential for higher education to proactively consider a range of supportive strategies (Cardel et al., 2020).

## Conclusions

The COVID-19 pandemic was not just a global health crisis; it was and is a globally shared life-altering crisis with long-term impacts on all sectors of life. While higher education is intended to serve as the “great equalizer” to better the lives of the next generation, (Chetty et al., 2017) it is also a historically gendered and racialized institution (Britton, 2000) that must vigilantly attend to the needs of its most vulnerable students, faculty, and staff. We used institutional data from one high research activity (R2) university and one very high research activity (R1) university to perform a self-study on our faculty’s decision making. Results illustrated patterns of differences in who accepted the stop-the-clock option and who did not, which we used to outline possible downstream implications that could exacerbate pre-existing disparities. With all eyes on pandemic mitigation efforts, it is easy to lose sight of the invisible and long-term ramifications. Offering multiple equity-oriented supports (Gonzalez & Griffin, 2020) and keeping diversity, equity, and inclusion at the forefront (Goodwin & Mitchneck, 2020) are essential to flatten the “inequity curve.”

## Data Availability

Available upon request.
